# Near-Field Generation and Control of Ultrafast, Multipartite Entanglement for Quantum Nanoplasmonic Networks

**DOI:** 10.1021/acs.nanolett.1c04920

**Published:** 2022-04-01

**Authors:** Frank Daniel Bello, Nuttawut Kongsuwan, Ortwin Hess

**Affiliations:** † School of Physics and CRANN Institute, Trinity College Dublin, Dublin 2, Ireland; ‡ Quantum Technology Foundation (Thailand), 98 Soi Ari, Bangkok 10110, Thailand; § Thailand Center of Excellence in Physics, Ministry of Higher Education, Science, Research and Innovation, Bangkok 10400, Thailand; ∥ Blackett Laboratory, Department of Physics, Imperial College London, London SW7 2AZ, United Kingdom

**Keywords:** Multipartite entanglement, quantum networks, quantum Internet, tripartite, near-field transducer, plasmonic waveguide, quantum dot, Greenberger−Horne−Zeilinger (GHZ) state

## Abstract

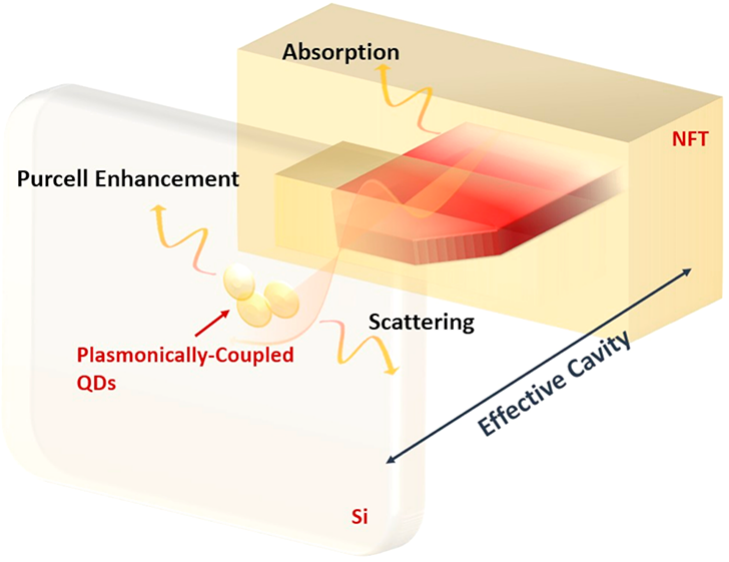

For a quantum Internet, one needs reliable sources of entangled particles that are compatible with measurement techniques enabling time-dependent, quantum error correction. Ideally, they will be operable at room temperature with a manageable decoherence versus generation time. To accomplish this, we theoretically establish a scalable, plasmonically based archetype that uses quantum dots (QD) as quantum emitters, known for relatively low decoherence rates near room temperature, that are excited using subdiffracted light from a near-field transducer (NFT). NFTs are a developing technology that allow rasterization across arrays of qubits and remarkably generate enough power to strongly drive energy transitions on the nanoscale. This eases the fabrication of QD media, while efficiently controlling picosecond-scale dynamic entanglement of a multiqubit system that approaches maximum fidelity, along with fluctuation between tripartite and bipartite entanglement. Our strategy radically increases the scalability and accessibility of quantum information devices while permitting fault-tolerant quantum computing using time-repetition algorithms.

With quantum computation steadily progressing beyond its infancy, research is presently focused on noisy intermediate-scale quantum (NISQ) computers^1^ and their inclusive networks. NISQ computers, comprised of roughly 50–100 qubits, are seen as the next-generation of computational technologies that will provide solutions to more complex problems in particular when combined with classical computing and at a vastly faster rate compared to present day supercomputers.^2,3^ However, it is crucial to consider the physical, societal, and environmental accessibility of such technology, and who can acquire it. To reduce noise, multiqubit quantum logic gates are often realized with superconducting quantum interference devices (SQUIDs) that require cryogenic confinement in highly specialized laboratories.^3^ These devices typically range from a few to tens of qubits that cannot be operated near room temperature. The noise and other decoherence effects created at higher temperatures is seen as detrimental to present-day, SQUID-based quantum networks. Nevertheless, decoherence and ultimate dephasing will naturally occur in many NISQ designs and may prove to be advantageous given that real-world systems, along with the many questions we wish to answer about them, are affected by decoherence.^4^ Recently, photonic-based quantum computers are attempting to achieve supremacy at room temperature with successful testing of small-scale classical computations, for example, squeezed states as quantum bits, that is, qubits, with scalability still to be determined.^5,6^ Herein, we embrace the noise and eventual dephasing of the network by using plasmonically coupled quantum dots, or analogously vacancy centers, as qubits. Such quantum emitters are seen as a pathway to move future quantum devices toward room temperature that are widely available, considerably more affordable, as well as scalable for NISQ designs and beyond.^7−9^

We verify those expectations by generating and controlling QD entanglement using relatively fast dephasing rates (10^11^ s^–1^), many orders of magnitude larger compared to SQUIDs,^10^ that we show are manageable from an experimental perspective, all the while keeping technological scalability in mind. To achieve this, we illustrate that multipartite entanglement can be manipulated using an effective open-cavity design by means of a near-field plasmonic transducer (NFT, schematic in Figure 1). For the first time, the NFT is shown to adeptly control the dynamic modulation of entanglement on the picosecond scale and create signal envelopes where tripartite and/or bipartite entanglement can be realized. This ultimately creates opportunities for error correction using repetition of experiment or code,^11^ thus paving the way for more fault-tolerant quantum computers as industries move toward a quantum Internet. A quantitative analysis of the full spatiotemporal dynamics of the system is performed (see Supporting Information for details) that shows a time-repeated fidelity greater than 0.99 (maximum value of 1) for the production of genuine multipartite entanglement (GME) in a 3-qubit (tripartite) system. Multipartite entanglement offers a wide range of applications not directly accessible to bipartite (2-qubit) entangled systems such as measurement-based quantum computation (MQC) with cluster states^12^ as well as quantum communication between multiple users and locations.^13^

**Figure 1:**
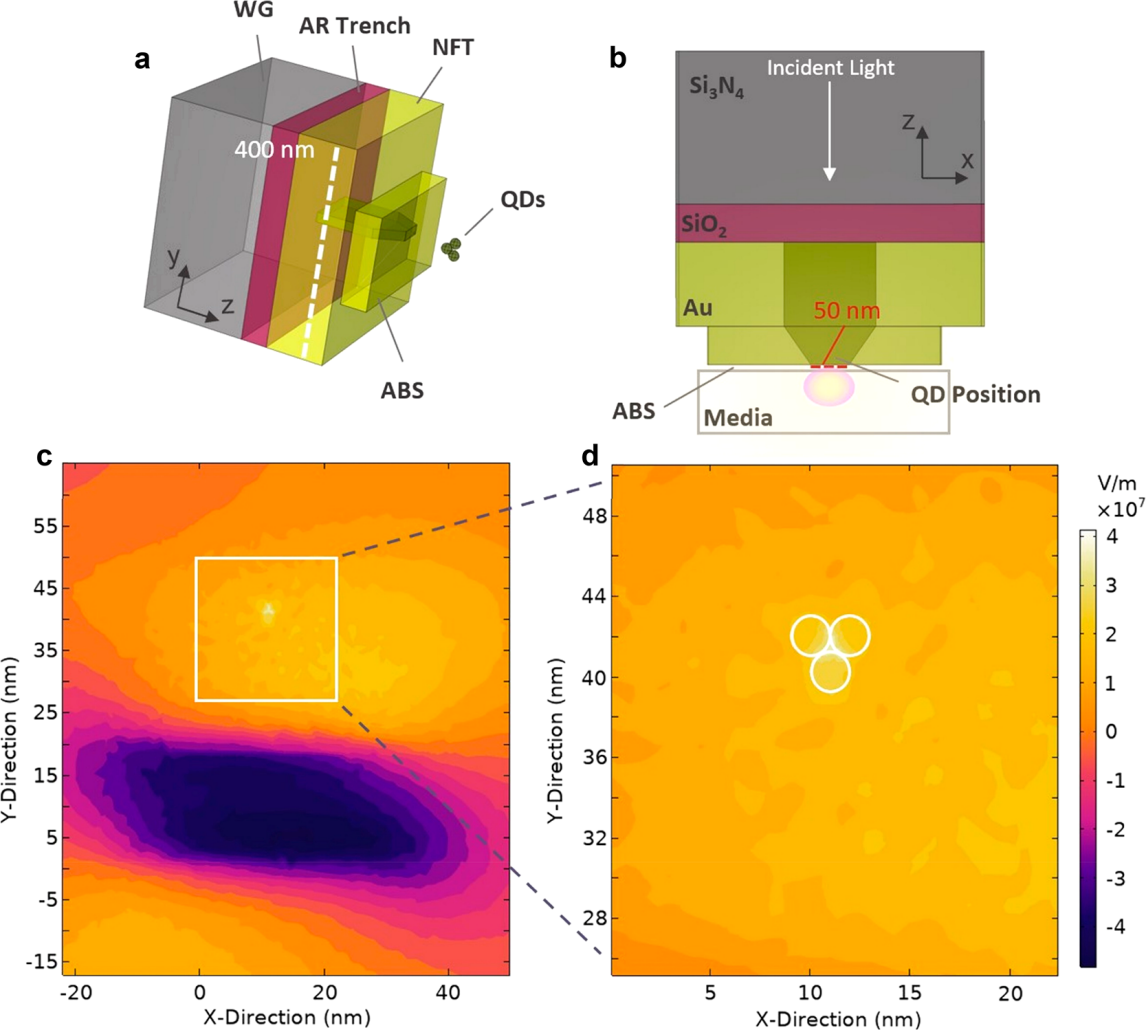
(a,b) A to-scale, minimal model of the NFT used to subdiffract light on the nanoscale along with three quantum dots. The single mode, silicon nitride waveguide (WG) excites an antisymmetric surface plasmon mode at wavelength of 830 nm in the NFT composed of Au and tapered SiO_2_ layer for nanofocusing. The light then couples to Si-based QDs of 2 nm in diameter and centered at 1.5 nm below the surface boundary of the QD media (see Supporting Information for alternative positioning). An antireflective (AR) trench composed of the same material as the tapered insulator (SiO_2_) is used to improve optical efficiency. (c) Steady-state field, Re(Ez), that couples to the QD dipole moments aligned in the *z*-direction. It is slightly skewed (to the right) rather than symmetric due light interacting with the QDs. (d) Contouring and overall strength of light scattered at the QDs’ position is highlighted. The 0-point on the figures represents the same position as the lower corner of the tapered insulator roughly centered in the NFT (see Supporting Information for parameter list).

To date, plasmonic meta-structures have shown considerable promise, and therefore attract much research effort, given their ability to reach ultrahigh-Q (quality) factors and be fabricated with advanced manufacturing techniques.^14−16^ Examples of such structures include a variety of cavities, 2D films, nanoparticles, and often a combination thereof with applications ranging from energy conversion to quantum information technologies.^17^ Plasmonic devices have the ability to subdiffract light on a scale of tens of nanometers by coupling the light to a surface plasmon, that is, a collection of oscillating surface charges, that is confined to a surface or boundary as it propagates. This notably replaces the use of a physical cavity in the QD media. Plasmon generation and eventual confinement of the near field is frequently achieved by using nanoplasmonics structures such as dimers or with nanoparticle-on-mirror (NPoM) geometries.^18,19^ However, these structures are often not movable, sometimes etched with bulk materials, or they need to be carefully placed in position using external, nonintegrated components. A plasmonic NFT is separate from the media containing the qubits, yet fully integrated with a photonic waveguide.^20^ It is therefore able to raster across a surface/media composed of quantum emitter arrays lying only a few to tens of nanometers away from the throughput end of the NFT. Altogether, they can reduce scenarios that cause damage to the emitter(s) or alter their initialized states while helping to keep an operating device closer to ambient temperatures. Moreover, NFTs have been pegged for use in the next generation of data storage devices with mass production for commercial use anticipated by 2022.

The schematic in Figure 1 depicts the metal–insulator–metal (MIM) NFT we consider along with sample dimensions, markedly larger than many NPoM structures and with the maximum intensities confined over regions under 50 × 50 nm.^2,20^ Optical energy efficiency, that is, percentage of input power delivered to the QD quantum emitters, is over 20%, thus comparable with similar state-of-the-art designs today.^21^ We emphasize that the electric or magnetic field may be enhanced, thus opening up magnetic/spin transitions, as a crucial alternative to our archetype (see Supporting Information for details).^22^ Importantly, the area of highest intensity is adjustable depending on input power and taper size at the air bearing surface and therefore capable of entangling multiple qubits simultaneously. This is compared to other, more narrowly tapered resonator/probe designs such as those used in atomic force microscopy.^21^ We build on the recent demonstration that NFTs are valid sources of subdiffracted, near-field light-generating sufficient field strengths incident on the QD media, as shown in Figure 1c,d, for near-field strong coupling, single-photon emission, enhancement of spontaneous emission, and bipartite entanglement.^23,24^ We now verify via theoretical simulation the ability to dynamically control multipartite entanglement on a picosecond scale while achieving excellent fidelity of the state we wish to excite. Thus, a much broader set of applications of NFT-based, near-field quantum dynamics for quantum communication is available.

In particular, by strongly driving the system we are able to control the rate that we produce entangled particles and the overall size of the dynamic envelope that includes the entanglement signal. Indeed, for a strongly driven system the energy mode splitting, defined by *g* = μ·*E*(*r*), must be greater than the modal decay rate, where μ is the dipole moment of the QD and *E*(*r*) is the scattered electric field at the QD’s position. A reasonable decay rate (γ_*r*_ = 1.2 × 10^11^ s^–1^) for plasmonically coupled QD-light systems estimated near room temperature, along with approximate values of the electric field on the order of 1 × 10^8^ V/m from Figure 1, yields *g* ≈ 1 × 10^13^ s^–1^ for a dipole moment of a few Debye. that is, roughly 2 orders of magnitude greater than the decay rate. We therefore underscore the use of NFTs in experiments that desire strong coupling, although the QD-NFT system proposed could be adjusted if a more weakly coupled system is preferred.

## Tripartite Entanglement

**Figure 2:**
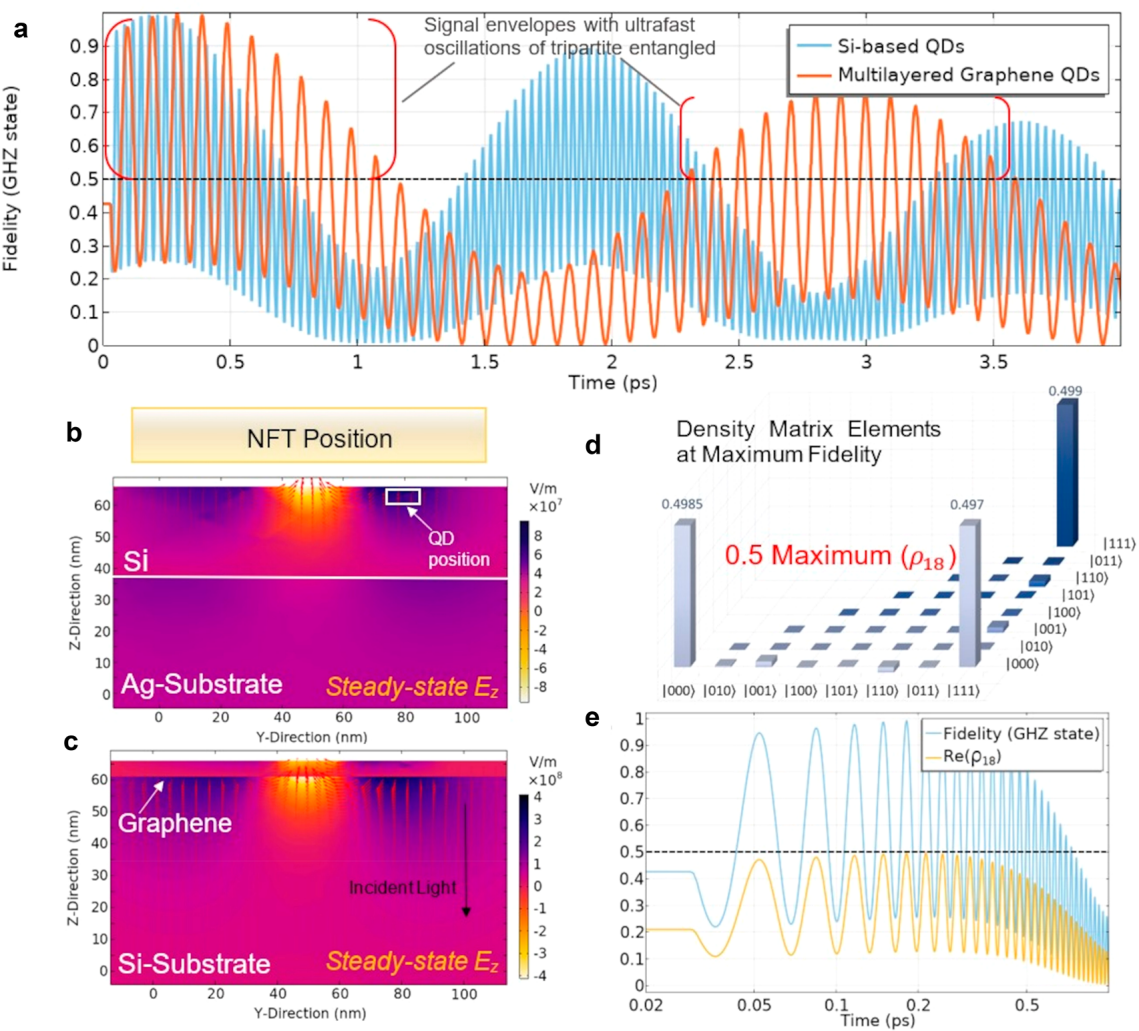
(a) Fidelity is shown for the production of the GHZ state in Si-based QDs (blue curve) along with QDs in multilayered graphene (orange curve) where tripartite entanglement occurs for values over 0.5. Both cases consider a dipole moment of 25 D. Signal envelopes (demarcated by red brackets) can be used for performing repetition experiments or algorithms for quantum error correction. A full parameter list for materials and physical dimensions is reported in the Supporting Information. (b,c) Steady-state field strengths are found on the order of 10^8^ V/m while keeping the temperature of Au between 400–450 K (see Supporting Information for calculation of temperature). Arrows represent the strength and direction of the displacement field with the NFT position lying above the profiles. (d) Real components of the density matrix elements (ρ_*ij*_ = ρ_ji_^*^) are shown corresponding to a maximum fidelity of 0.993 for Si-based QDs at a time of 0.213 ps and reveal excellent control of the GHZ state with imaginary components ≤|0.05|. (e) Correspondence between the coherence term, ρ_18_, of the GHZ state and fidelity is shown with values approaching the maximum value of 0.5. The incident pulse is turned on at 0.03 ps.

We investigate the 3-qubit model outlined in Figure 1 and use a system composed of identical 2-level quantum dots, analogous to many spin-1/2 systems. Considering experimentally accessible initial conditions and material sets (see Supporting Information for full details), our analysis includes Si-based quantum dots that may also be characteristically embedded in multilayered graphene^25^ (5 nm), desirable given its properties of optical conductivity. In Figure 2, we report the statistics on the fidelity of entanglement using the density matrix formalism to describe the QD dynamics that quantitatively is coupled to the electric field in space and time using Maxwell’s equations (see Supporting Information for details of theoretical methods). Multipartite entanglement in the system occurs by manipulating the excitation of the Greenberger–Horne–Zeilinger (GHZ_3_) state, that is, (|000⟩ + |111⟩)/√2, and inducing a fidelity, defined as 1/2(ρ_11_ + ρ_88_ + *C*), over 0.5^3^. Here *C* = 2|ρ_18_| is dependent on the coherence term between the ground (|000⟩) and excited (|111⟩) states of all three QDs with ρ_11_ and ρ_88_ the populations of each, respectively. The QDs are considered to be on resonance with the incident pulse and positioned such that they are approximately aligned with the maximum field intensity as shown in Figure 1. The QD diameter is set at 2 nm and centered 1.5 nm from the media surface. It is remarkable to note the relative ease of manipulating the entanglement of the multipartite system by using the NFT to simultaneously drive the dipole moments with the plasmonic field. No particular film size is required. Tripartite entanglement is dependent on the oscillation strength which in turn may be controlled by the dipole moment strength or the embedding media which affects the absorbed power. This control is shown in Figure 2a by switching from QDs embedded in silicon to QDs embedded in multilayered graphene. A sustained repetition of tripartite entanglement (fidelity >0.5) with fidelities approaching the maximum of 1, for example, 0.998 for graphene-based QDs and 0.993 for Si-based QDs, are achievable in both cases with possibilities for improvement. This enables the implementation of error correcting experiments or algorithms, that is, repetition codes, performed over signal envelopes emitted over the bracketed time periods, as highlighted (red brackets) for the case of QDs in graphene. The change in absorbed intensity can be seen in Figure 2b,c depending on the material in which the QDs are embedded, while in Figure 2d we show the coherent control of the GHZ state by plotting the density matrix elements and spotlight the correspondence of the fidelity with that of the coherence term for the GHZ state in Figure 2e. Changes in QD position or film size may also change the rate of oscillations and prove beneficial for optimizing a particular design.

**Figure 3:**
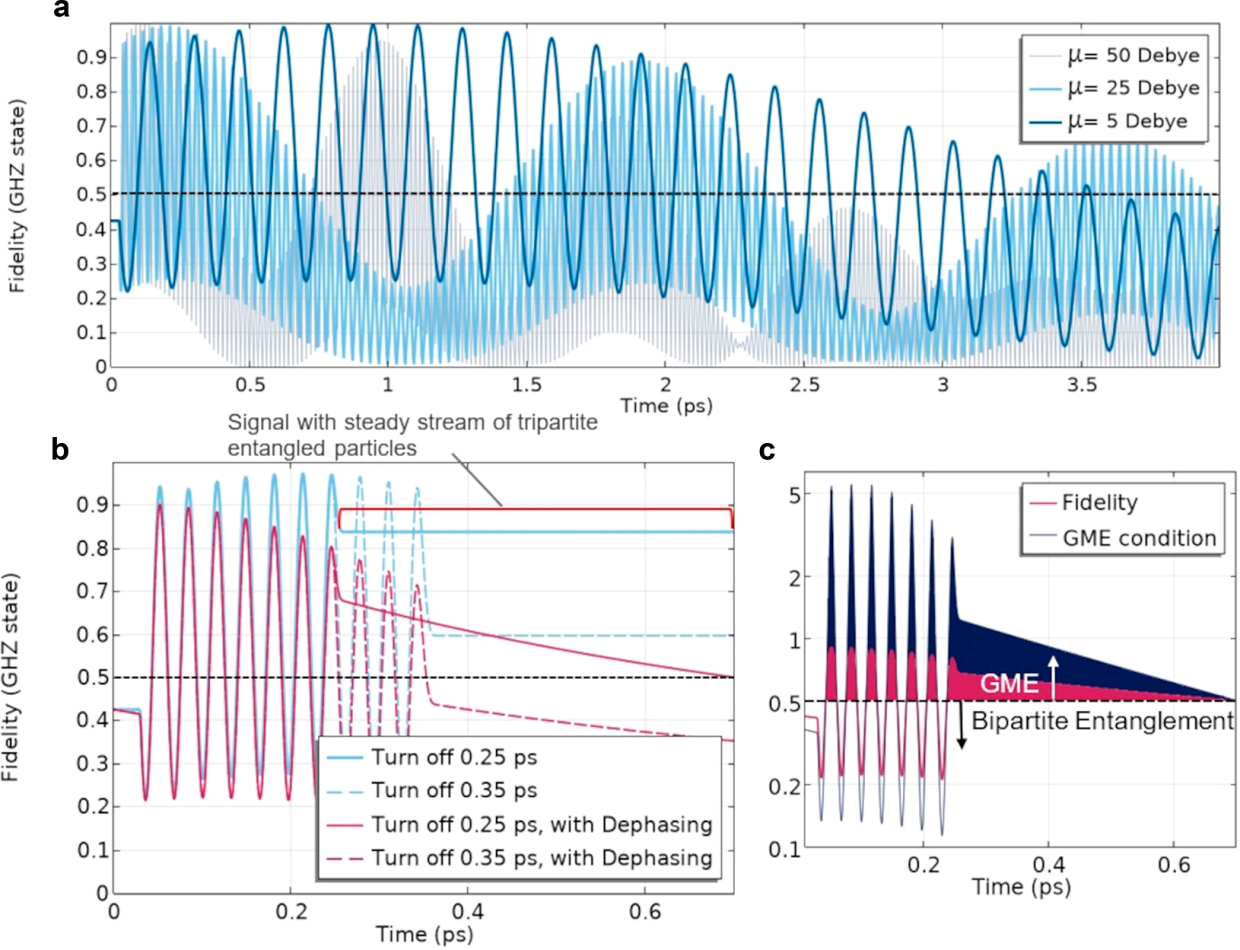
(a) We have plotted the fidelity for the GHZ state (tripartite entanglement for values >0.5) when examining a number of different dipole moment (μ) strengths for Si-based QDs. By varying μ, we are able to elongate or shorten the oscillation rate of the fidelity curve. Values ranging from 5–50 D are readily achievable in strongly coupled systems and in particular those that use graphene or transition metal dichalcogenides (TMDCs).^25^ (b) When we turn off the pulse at various times, we are able to induce a steady stream of tripartite entangled particles (blue curves, example demarcated in red bracket). Even when decay and dephasing rates, γ_*r*_ and 4γ_*r*_, respectively, are included (purple curves) the steady stream lasts for many hundreds of femtoseconds. (c) The condition for genuine multipartite entanglement (GME, filled region) is shown for the case in Figure 2b with the power turned off at 0.25 ps. It agrees with and is equivalent to the tripartite entanglement condition in our case from the fidelity (both greater than 0.5) and corroborates alternation between tripartite entanglement (GME) and bipartite entanglement in all cases (see Supporting Information for details).

The effects of adjusting the dipole moment may be seen in Figure 3a which shows near maximum fidelity for a number of values that are typically dependent on the level of coupling between light and the QD. Values ranging between 5 and 50 D are readily achievable in coupled systems^26^ with a smaller (larger) dipole moment able to lengthen (shorten) the oscillation rate of tripartite entanglement. Remarkably, a consistent production of multipartite entangled particles is maintained in each case with similar envelope behavior (see Supporting Information for entanglement behavior up to 20 ps). Moreover, by turning off the laser pulse at a particular time^27^ we show that one has the ability to manipulate a steady stream of entangled particles. Under this scenario, all effects from the NFT and therefore the open cavity system, are removed from the simulation. Figure 3b illustrates how a nonoscillating, steady state of tripartite entanglement may be achieved under conditions of extremely low or no dephasing (cryogenic system, blue curves). The effects of dephasing (purple curves) are included as well and affirm the stable production of tripartite entanglement over hundreds of femtoseconds. In fact, genuine multipartite entanglement (GME) is achieved with the entangled GHZ state, defined when , such that the 3-qubit system is not entangled if the density matrix is reduced, that is, it cannot be separated into two entangled qubits and an unentangled third particle.^28^ This condition is met and depicted in Figure 3c when the GME condition >0.5 (filled regions) in agreement with the requirement on fidelity when tripartite entanglement is achieved. We note that bipartite entanglement (unfilled regions) is predicted when the GME condition <0.5 and exists between two states within the full unreduced density matrix.^28,29^ Therefore, controlled alternation between bipartite and tripartite entanglement is realized, which was recently confirmed to be attainable in non-Hermitian parity-time (PT) symmetric coupled cavity systems^30,31^ along with inherent applications toward quantum teleportation.

### Adjustments to Decay/Dephasing Rates

**Figure 4:**
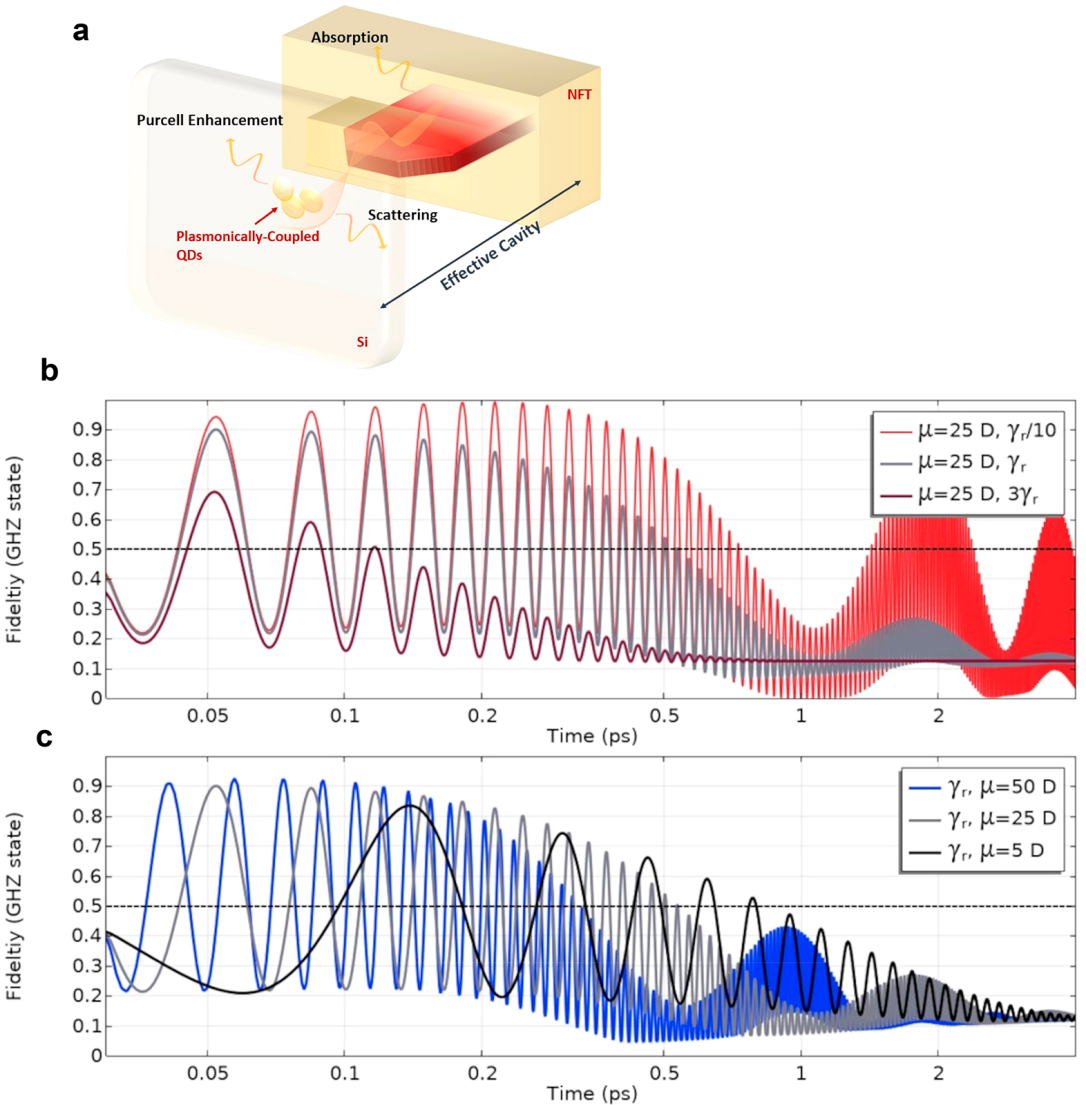
(a) Schematic of the effective open-cavity system consisting of the NFT which propagates light via an SPP mode that subsequently couples to the QDs placed in separate media. We emphasize several contributions to the decay and dephasing rates such as the Purcell enhancement (radiative) along with scattering or absorption loss. Altogether, these are anticipated to yield ultrafast (picosecond-scale) lifetimes which we use in our investigation.^32^ Various fabrication techniques, along with a number of possible NFTs, can be used to produce nanofocused light. All of which can lead to changes to the decay (γ_*r*_) and dephasing rates (4γ_*r*_) in the NFT+QD system. (b) We assume some variations to the radiative decay rate used (γ_*r*_ = 1.2 × 10^11^ s^–1^) with good fidelity noticed for decay rates as high as 3γ_*r*_ (dephasing = 12γ_*r*_). (c) Depending on the dipole moment, the time period that one sees oscillations with fidelities above 0.5, that is, multipartite entanglement, can be extended using a lower strength, for example, that of 5 D. Fidelities could be increased for stronger moments, such as 25 or 50 D, where more rapid oscillations are shown to occur and anticipated given the faster Rabi oscillation rates for larger dipole moments. For the scenario presented, GHZ entanglement disappears when the decay rate is increased 5× (5γ_*r*_) with dephasing rates also increased to 20γ_*r*_ (2.4 × 10^12^ s^–1^).

The schematic in Figure 4a represents the effective open-cavity system used, which consists of light from surface plasmon polaritons (SPPs) produced by the NFT that is coupled to the QDs in separate media.^21^ Given the effective cavity system, decay and especially dephasing rates are anticipated to vary. The decay rate includes enhancements to the spontaneous emission rate, that is, Purcell enhancement, expected to be on the order of 10^2^–10^3^ depending on materials used.^32^ This enhancement has been experimentally measured for plasmonically coupled QDs that yielded decay rates on the inverse picosecond scale, which we have included throughout our investigation, although variations to the exact decay/dephasing rates for our system may indeed occur. For example, absorption loss through any metallic components in particular as well as scattering loss within the media contribute to ultrafast lifetimes. Figure 4b displays how changes to the radiative decay (γ_*r*_) and dephasing rates (4γ_*r*_) could affect the GHZ entanglement. Very good fidelities are still calculated (>0.7) using decay rates 3× faster (12× faster for dephasing) than the original values. We note GHZ entanglement is attainable for faster decay processes if dipole moments are increased. For example, multipartite entanglement is noticed (fidelity = 0.516) for dipole moments of 50 D using 5× (5γ_*r*_) the original decay rate with dephasing rates also increased to 20γ_*r*_. Stronger dipole moments are also able to increase the oscillation rates of the NFT+QD system in and out of multipartite entanglement. If desired, by using lower dipole moment strengths, such as 5 D shown in Figure 4c, one can maintain slower Rabi oscillation rates and thus extend the time scale over which GHZ lasting entanglement though fidelities are slightly lower. It should also be noted that by reducing the quality of the effective cavity, which may be done either by reducing the field confinement or using a lower dipole moment, one can lessen the Purcell enhancement if emission rates become too large and therefore extend the time period that GHZ entanglement will last.

To conclude, this study verifies using theoretical simulation the enormous potential of NFT-promoted, near-field nanoplasmonics for generating and controlling multipartite entanglement, which includes time-dependent dephasing effects appropriate near room temperature. The ability to adeptly manipulate quantum superposition and entanglement that includes significant dephasing on a nano and ultrafast scale is necessary to perform more efficient, fault-tolerant operations for use in on-chip, multipartite quantum devices. Efficient and effective control of entanglement has been shown using an effective open-cavity QD-NFT system excited with near-field, nanoscale-focused light from the NFT. Moreover, the archetypal QD-NFT system opens up possibilities to perform multipartite quantum logic gates, such as the Toffoli gate, given the control over QDs and moveability of an NFT structure. NFTs are a relatively new technology only recently verified to strongly drive QD systems^24^ but now with the added ability to proficiently manipulate multipartite entanglement with optimum fidelity and provide added rastering capability. Crucially, this generates sufficient entanglement before dephasing rates decohere the system. Genuine multipartite entanglement using a 3-qubit, tripartite system was shown to occur with an excellent fidelity of entanglement approaching 1 for excitation of the GHZ state. Furthermore, ultrafast control over entanglement (tripartite or bipartite) is achievable using advanced manufacturing techniques that control dipole moments, embedding media, incident power, and initial conditions.^33^ These advancements greatly improve the compactness and accessibility of quantum information devices given the ability to couple light on the nanoscale, opening up major implications for storing quantum memories as well as performing algorithms with higher fidelities.

Additionally, QDs, along with other emitters, show promise for quantum information processing at conditions closer to room temperature given their dephasing rates, while also being spatially flexible^34^ given their ability to be created and erased on demand.^35^ Overall, these projections move NISQ concepts for quantum networks forward while using scalable, nanoscale designs which are operable near room temperature.

## Supporting Information Available

Parameter list for materials used; parameter list for NFT dimensions; methods used in simulations; description of the density matrix; incident field power; extended time simulation of fidelity; initial conditions used; examples of density matrix elements; sample mesh of elements used in the FETD simulation; methods and calculations of device temperature (PDF)

## ABBREVIATIONS

GHZ: Greenberger–Horne–Zeilinger
QD: quantum dot
NFT: near-field transducer
NISQ: noisy intermediate-scale quantum
SQUID: superconducting quantum interference devices
GME: genuine multipartite entanglement
MQC: measurement-based quantum computation
NPoM: nanoparticle-on-mirror
MIM: metal–insulator–metal
FETD: finite-element time domain;
