# Supplementary material for: Near-Field Generation and Control of Ultrafast, Multipartite Entanglement for Quantum Nanoplasmonic Networks
Source: Nano Lett. 2022 Apr 1;22(7):2801–8. doi: 10.1021/acs.nanolett.1c04920 (PMC9011391; doi:10.1021/acs.nanolett.1c04920)
Supplement: Supplementary file 1 — nl1c04920_si_001.pdf [file nl1c04920_si_001.pdf]

Near-Field Generation and Control of Ultrafast, Multipartite Entanglement for Quantum Nanoplasmonic Networks

Frank Daniel Bello[1], Nuttawut Kongsuwan[2,3,4], Ortwin Hess,[1,4*]*

[1]School of Physics and CRANN Institute, Trinity College Dublin, Dublin 2, Ireland
[2]Quantum Technology Foundation (Thailand), 98 Soi Ari, Bangkok, 10110, Thailand
[3]Thailand Center of Excellence in Physics, Ministry of Higher Education, Science, Research and Innovation, Bangkok, 10400, Thailand
[4]Blackett Laboratory, Department of Physics, Imperial College London, London, SW7 2AZ, UK
*Corresponding authors: bellofr@gmail.com, ortwin.hess@tcd.ie

Supplementary Information:

Theoretical and Simulation Methods

Finite-element time domain (FETD) simulations were used to derive the electric field incident on the QD media by solving the full-field, steady state solution of Maxwell's equation for the photonic waveguide and MIM-NFT resonator shown in Figure 1 of the main text, which lies above the media stack[S1]. The photonic waveguide (2 μm long) is considered to be under a peak input power of 17 mW at a wavelength of 830 nm with approximately 24% of maximum power reaching the QD layer. This input power is able to keep the temperature of the Au at approximately 400 K, which is desired to prevent protruding of the metal and potential damage to the NFT. If desired, lower temperatures may be achieved using various metal alloys. The media is initially composed of Air/lubricant (2 nm) and an Si-rich QD layer (29 nm) on an Ag substrate. Variations to the material used for the QD layer is found to keep the coupling field strength on the order of 10^8 V/m,

which may be compensated for by adjusting the input power, and therefore for consistency we use the same input field throughout the study. We then separately solve for the time-dependent density matrix of the 3-qubit system for spin-½ QDs (8×8 matrix) and remove the waveguide core and MIM resonator from numerical simulations considering the incident electric field as their primary contribution to the dynamics of the QD system. This method has been effectively used to model the power dissipation and heat transfer dynamics in similar NFT + media systems.

The effect the QDs have on their surrounding environment is included within Maxwell's equations by computing the polarization induced by their dipole moment, where all variables are a function of time and 3-dimensional space.

$$\varepsilon \partial_t \mathbf{E} = \nabla \times \mathbf{H} - \partial_t \mathbf{P}, \tag{S1}$$

$\mathbf{P}$ is calculated at the QD's position ($\boldsymbol{r}_i$) along with the ensemble averaged polarization ($\mathbf{p}$) which is defined as a function of the density matrix ($\hat{\rho}$), $\mathbf{P} = \langle \mathbf{p} \rangle = \mathrm{Tr}(\mu \hat{\rho})$. The time-dependent density matrix is defined as a function of the Hamiltonian ($\hat{H}$),

$$i\hbar \partial_t \hat{\rho} = \left[\hat{H}, \hat{\rho}\right] - i\hbar \hat{\Gamma} \hat{\rho}, \tag{S2}$$

where $\hat{\Gamma}$ is the super operator that includes the decay and dephasing of each energy level. Here, we consider relatively fast decay/dephasing rates with dephasing four times that of the decay. Although they are not required, lower temperatures are known to improve these values. The Hamiltonian shown below uses raising and lowering energy level operators, $\sigma_i^{\pm}$, for a 3-qubit ($i \in 1-3$) system, with ω_i the frequency of the QD energy level. Of note, a coherent coupling term between quantum emitters is not explicit in the Hamiltonian, rather it is implicitly incorporated by the spatial integration of the displacement field throughout the media.

$$\hat{H} = \sum_{i=1-3} \omega_i \sigma_i^{+} \sigma_i^{-} + E_i^{sc}(\mu\sigma_i^{+} + \mu^{*}\sigma_i^{-}) \quad \text{(S3)}$$

We use a symmetry-adapted averaging approach of the electric field to exclude all self-interactions the QD may have with its emitted field[S2]. Therefore, the scattered field used in the Hamiltonian is defined as an average of the full field solution in the region of the QD, $E_i^{sc} = \frac{1}{N}\sum_j \mathbf{E}(r_j)$, where '$N$' is the number of data points defined within the QD with the summation going from 1 to N. The FETD simulations utilize a free tetrahedral mesh (Shown in the supplementary information) with distances between data points ranging from 0.6 nm - 32 nm, maximum element growth rate of 1.5, and relative tolerances tested between 0.001-0.01. Density matrix elements are calculated with an error estimated on the order of 10^{-3}. A modestly shared coherence between states is found suitable for initialization of the system (See section on initial conditions).

The density matrix is formed by taking the tensor product for three, 2-level quantum dots (QD) defined by a wave function using the standard basis state, $|\Psi\rangle = \alpha|0\rangle + \beta|1\rangle$, for individual QDs with $\alpha\alpha^{*}$ and $\beta\beta^{*}$ the probabilities to be in either the ground or excited state, respectively. The tensor product is defined as $|\varphi\rangle = |\Psi_1\rangle \otimes |\Psi_2\rangle \otimes |\Psi_3\rangle$ and the density operator as $\hat{\rho} = |\varphi\rangle\langle\varphi|$ for the tripartite system. This yields an 8×8 matrix when applying the appropriate *bra* and *ket* notation with elements corresponding to the tripartite basis states $|000\rangle$, $|010\rangle$, $|001\rangle$, $|100\rangle, |101\rangle, |110\rangle, |011\rangle, |111\rangle$. The diagonal elements yield the populations of each level while the off-diagonal elements are the coherences that are necessary to calculate the polarization and fidelity. We then solve the density matrix equations while ensuring the trace $\sum_{i=1}^{4} \rho_{ii} = 1$ is conserved. The incident electric field profile is considered monochromatic and time dependent which yield the detunings, δ_{ij}, between each energy level and the laser. Parameters optimized for

the tripartite system are reported in Tables S1 and S2 while Figure S7 shows a to-scale schematic of the full photonic waveguide and NFT structure.

In addition to using the fidelity of the GHZ state to calculate tripartite entanglement, there is a condition for the coherence terms to generate genuine multipartite entanglement (GME) from a biseparable state as defined in Gühne et al[S3] and given by $|\rho_{18}| > \sqrt{\rho_{22}\rho_{77}} + \sqrt{\rho_{33}\rho_{66}} + \sqrt{\rho_{44}\rho_{55}}$. Satisfying this condition is equivalent to achieving tripartite entanglement, which we have plotted with the fidelity in Figure 3c of the main text, though we adapt the condition to the form,

$$\frac{|\rho_{18}|/(\sqrt{\rho_{22}\rho_{77}}+\sqrt{\rho_{33}\rho_{66}}+\sqrt{\rho_{44}\rho_{55}})}{2} > \frac{1}{2}, \tag{S4}$$

in order to match when the fidelity goes over 0.5. Values over 0.5 for fidelity demonstrate multiparticle entanglement, which the GME condition corroborates, with bipartite entanglement achieved between two states of the 3-qubit density matrix for values under 0.5 [S4].

Initial Conditions

The initial states used in our simulations are reported in the charts of Figure S1 that yield an initial fidelity of roughly 0.4, i.e. no initial multipartite entanglement. We find nothing particular about the initial conditions we use to produce tripartite entanglement except to have a shared coherence between coherence terms. In our case, the off diagonal elements have a modest coherence with amplitudes on the order of 0.2 or less that may be experimentally realized[S5]. Tripartite entanglement has also been successfully reproduced by setting the imaginary terms to zero, and even by further reducing the real terms by at least 10% for certain conditions. We have found these

Incident Electric Field and Density Matrix

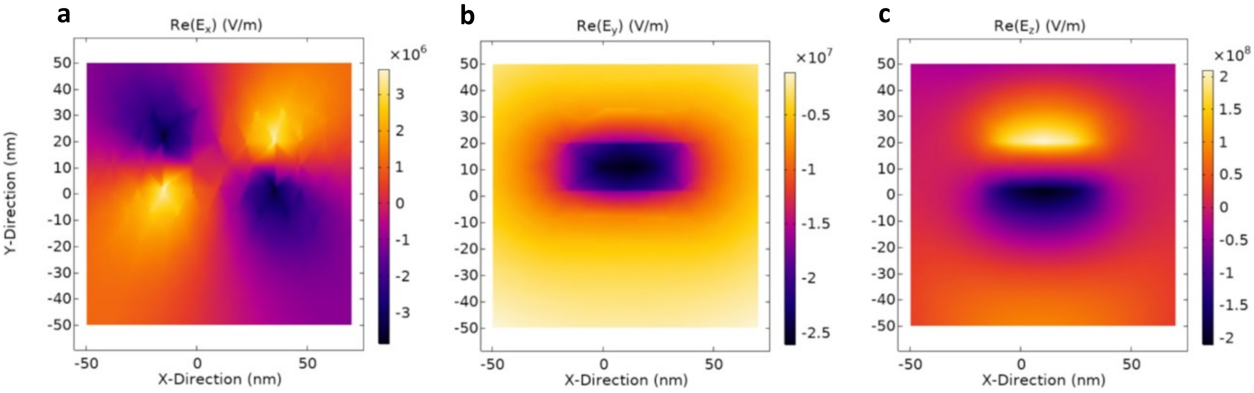

d

	$\|000\rangle$	$\|010\rangle$	$\|001\rangle$	$\|100\rangle$	$\|101\rangle$	$\|110\rangle$	$\|011\rangle$	$\|111\rangle$
$\|000\rangle$	0.2154	-0.0677	-0.0743	-0.1171	-0.1099	-0.0752	-0.0844	0.2096
$\|010\rangle$		0.0949	0.1068	0.0573	0.0586	0.1092	0.097	-0.0451
$\|001\rangle$			0.1201	0.0637	0.0652	0.1228	0.1089	-0.0489
$\|100\rangle$				0.0696	0.0666	0.0647	0.0656	-0.1083
$\|101\rangle$					0.0641	0.0663	0.0661	-0.1002
$\|110\rangle$						0.1256	0.1113	-0.0491
$\|011\rangle$							0.1005	-0.0623
$\|111\rangle$								0.2098

e

	$\|000\rangle$	$\|010\rangle$	$\|001\rangle$	$\|100\rangle$	$\|101\rangle$	$\|110\rangle$	$\|011\rangle$	$\|111\rangle$
$\|000\rangle$		0.1258	0.1425	0.0348	0.0408	0.1462	0.1203	0.0356
$\|010\rangle$			-0.0013	0.0576	0.0514	-0.002	0.0115	-0.1336
$\|001\rangle$				0.0655	0.0586	-0.0007	0.0143	-0.1509
$\|100\rangle$					-0.0045	-0.0674	-0.0518	-0.0531
$\|101\rangle$						-0.0604	-0.0454	-0.0578
$\|110\rangle$							0.0153	-0.1546
$\|011\rangle$								-0.131
$\|111\rangle$								

Figure S1. Real components (a)-(c) of the electric field incident on the surface of the QD medium are shown along with initial conditions for the density matrix, though these conditions are found to not be mandatory. Since our Hamiltonian is Hermitian, we have $\boldsymbol{\rho_{ij} = \rho_{ji}^{*}}$. The dipole moments of each QD are considered identical and aligned in the z-direction, though scattering effects may occur from the other components and hence a full 3D, spatiotemporal, FETD simulation is performed.

requirements on the shared coherence by setting an initial condition with tripartite entanglement and watching the system evolve in time to an unentangled 3-qubit state.

Although no magnetic transitions were utilized in our study, we highlight the magnetic fields that would otherwise be incident on the QD media in Figure S2. We note that components of the magnetic field may be enhanced and magnetic transitions, such as those in Diamond vacancy centers, can be used as an alternative quantum emitter.

Magnetic Field from Near-Field Transducer

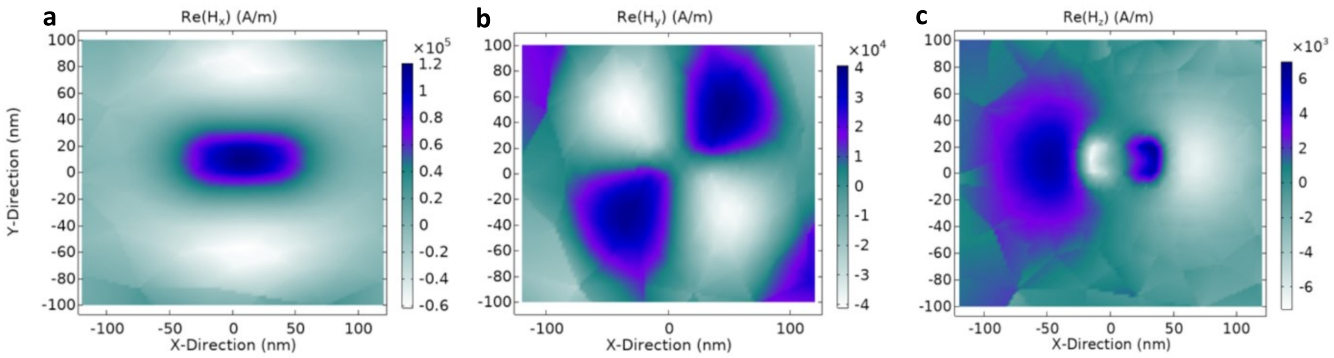

Figure S2. Real components (a)-(c) of the magnetic field are shown that would otherwise be incident on the QD medium though no magnetic transitions were utilized. Nonetheless, NFTs may be designed to enhance a particular component of the E- or H- field and we note that systems with magnetic transitions are viable for the proposed entanglement scheme. Maximum field strengths for H_x and H_y are roughly an order of magnitude greater than regions outside of the area shown while H_z has minimal enhancement.

Full Density Matrix Results

a

Real Density Matrix Elements for QDs in Multilayered Graphene at Max Fidelity (0.998)

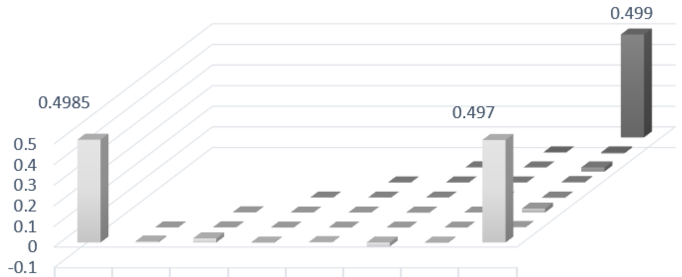

	|000⟩	|010⟩	|001⟩	|100⟩	|101⟩	|110⟩	|011⟩	|111⟩
|000⟩	0.4985	0.005	0.02	-0.002	0.002	-0.019	-0.003	0.497
|010⟩		0.0005	0.001	0	0	0	0	0.003
|001⟩			0.001	0	0	-0.001	0	0.019
|100⟩				0	0	0	0	-0.002
|101⟩					0	0	0	0.002
|110⟩						0.001	0	-0.02
|011⟩							0	-0.005
|111⟩								0.499

Imaginary Density Matrix Elements for QDs in Multilayered Graphene at Max Fidelity (0.998)

b

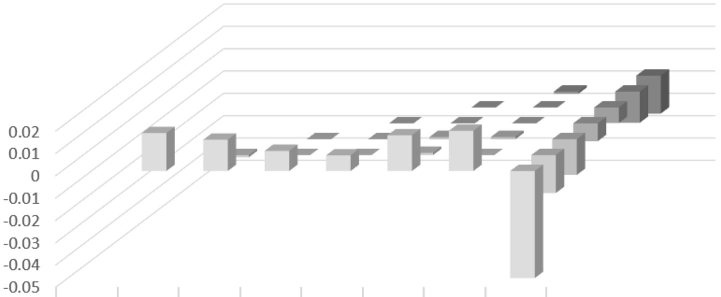

	|000⟩	|010⟩	|001⟩	|100⟩	|101⟩	|110⟩	|011⟩	|111⟩
|000⟩		0.017	0.014	0.009	0.007	0.016	0.018	-0.048
|010⟩			-0.001	0	0	0.001	0	-0.017
|001⟩				0	0	0.001	0.001	-0.016
|100⟩					0	0	0	-0.008
|101⟩						0	0	-0.007
|110⟩							-0.001	-0.014
|011⟩								-0.017
|111⟩								

Figure S3. Real (a) and imaginary (b) components are shown for the time of maximum fidelity (0.998) using QDs embedded in multilayered Graphene. Reported density matrix elements are rounded to the thousandth decimal place given an error on the order of 10^{-3} reported. Most of the coherence can be seen to pool in the GHZ state, $\boldsymbol{\rho_{18}}$, compared to the initial conditions in Figure S1 (d) and (e).

Fidelity Over 20 ps

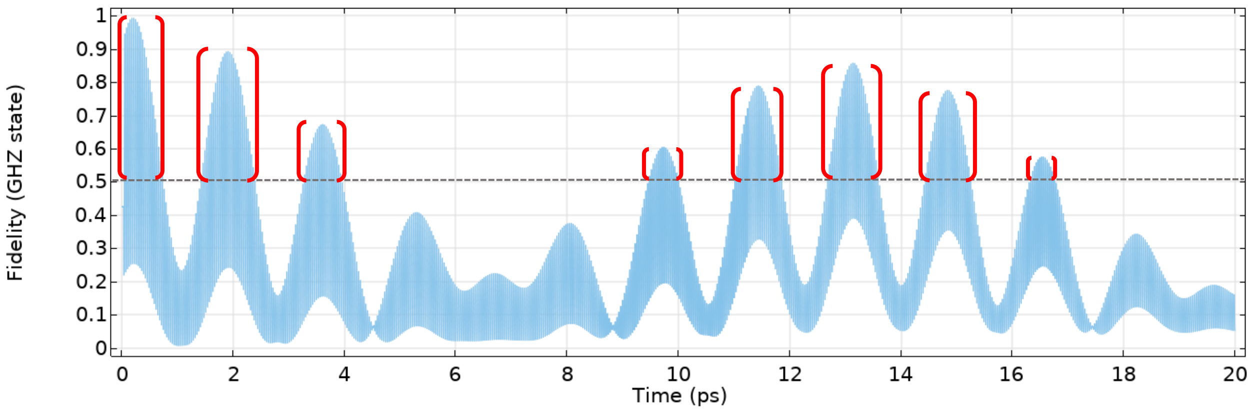

Figure S4. The fidelity is shown for QDs embedded in Silicon that is expanded from 4 ps (Figure 2a in the main text) to over a period of 20 ps. Decay/dephasing rates are neglected in this case. Repeated occurrences, i.e. signal envelopes (bracketed), of tripartite entanglement are demonstrated when fidelity is greater than 0.5. Below 0.5, bipartite entanglement exists from the GME condition of Eq S4. Each signal envelope that contains particles oscillating in and out of tripartite-bipartite entanglement provides opportunities for quantum error correction via repetition experiments or code implementation.

Position of Quantum Emitters

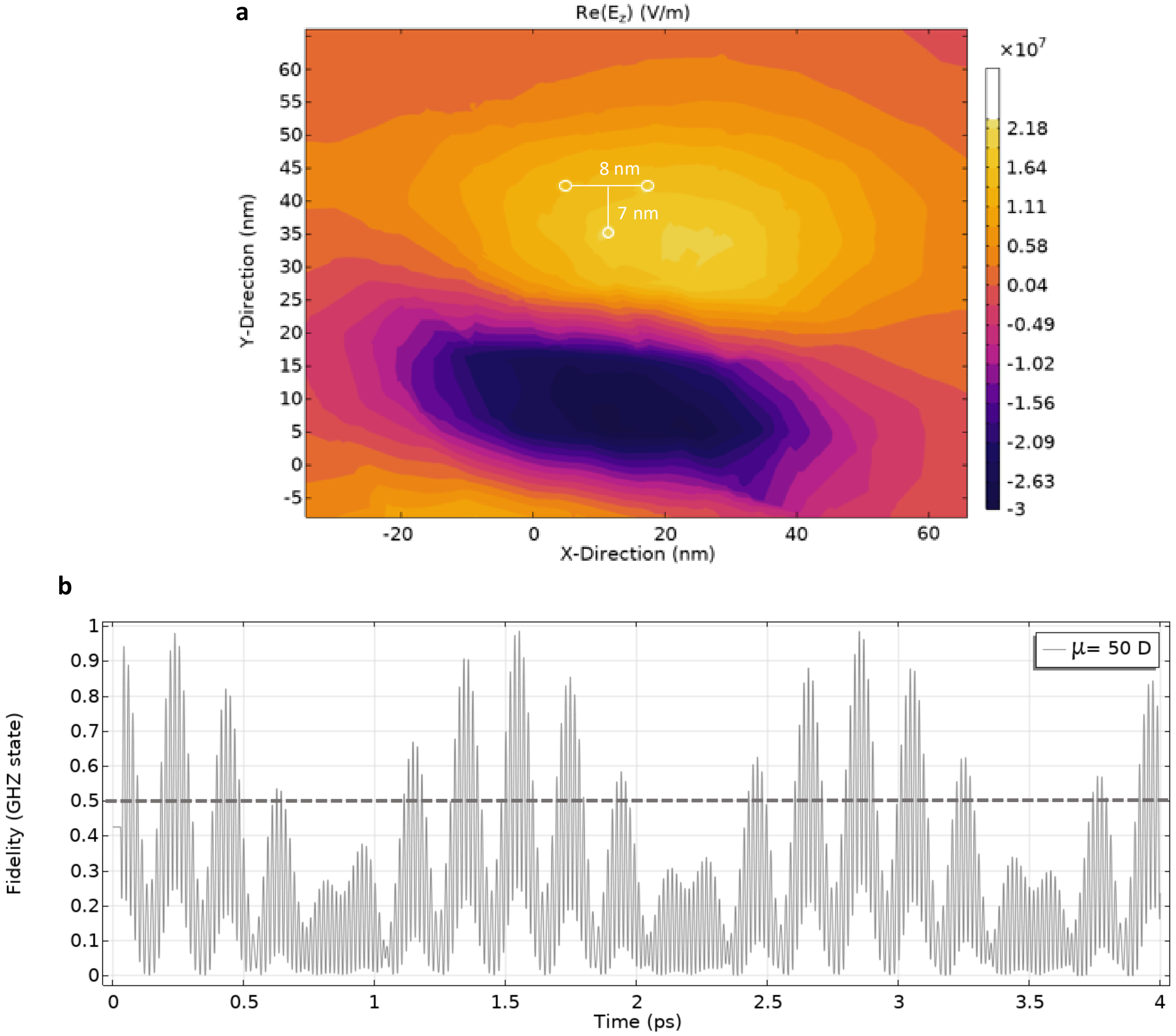

Figure S5. (a) Depending on the type of quantum emitters used, electrical coupling may occur and hence some separation of QDs may be desired. We demonstrate an example that shows comparable results for multipartite entanglement (Max Fidelity = 0.984) using 3 quantum dots with a dipole moment strength of 50 D each removed an additional 5 nm from their original position in the main text. (b) We note that by changing the position of QDs, the size of the regions (signal envelopes) with fidelity greater than 0.5 may be controlled. This is an additional degree of freedom which may be optimized depending on experiment.

Input Fields

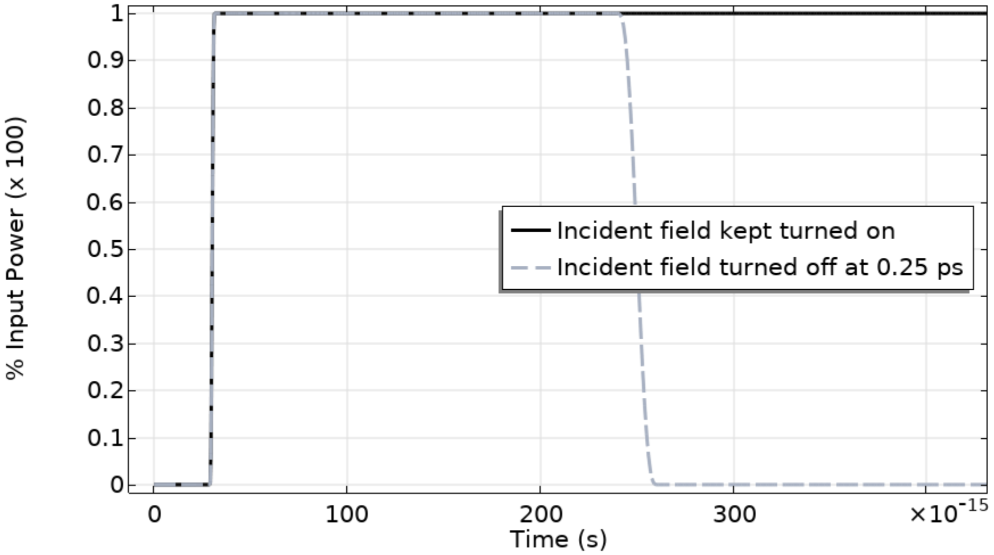

Figure S6. The percentage of input power is depicted as a function of time. It is turned on at 0.03 ps for all cases and turned off at either 0.25 ps (dashed curve) or at 0.35 ps for the cases reported in Figure 3b of the main text.

Free Tetrahedral Mesh for FETD modeling

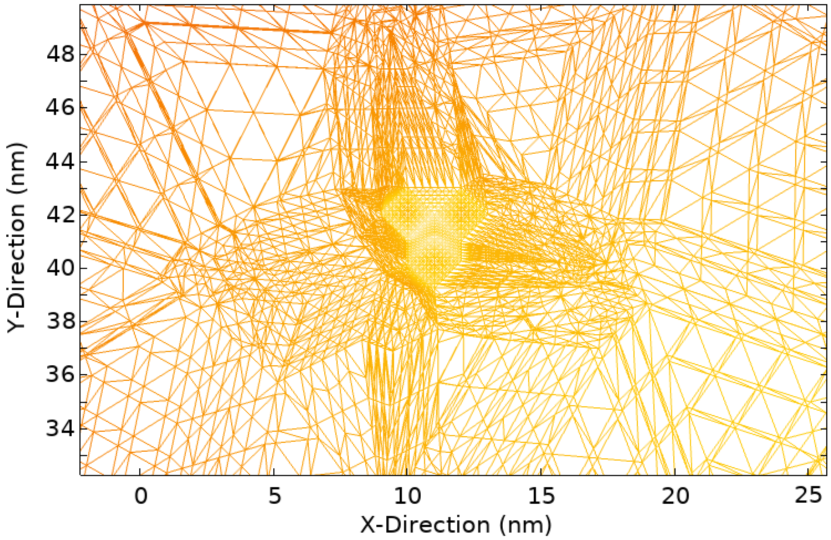

Figure S7 Here we highlight Figure 1d of the main text and show the density of the free tetrahedral mesh used, i.e. positions of data points collected throughout the structure. The figure focuses on the QDs where the densest part of the mesh is formed with elements as small as 0.6 nm to ensure convergence of Maxwell's equations and the density matrix. As we move away from the QD positions asymmetries in the mesh arise, which is typical when using conformal meshes which depends on mesh (tetrahedral in our case) and overall structure shape. Though it should be noted the field profile and shape matches well to previous simulations using symmetric meshes.

Temperature Calculations

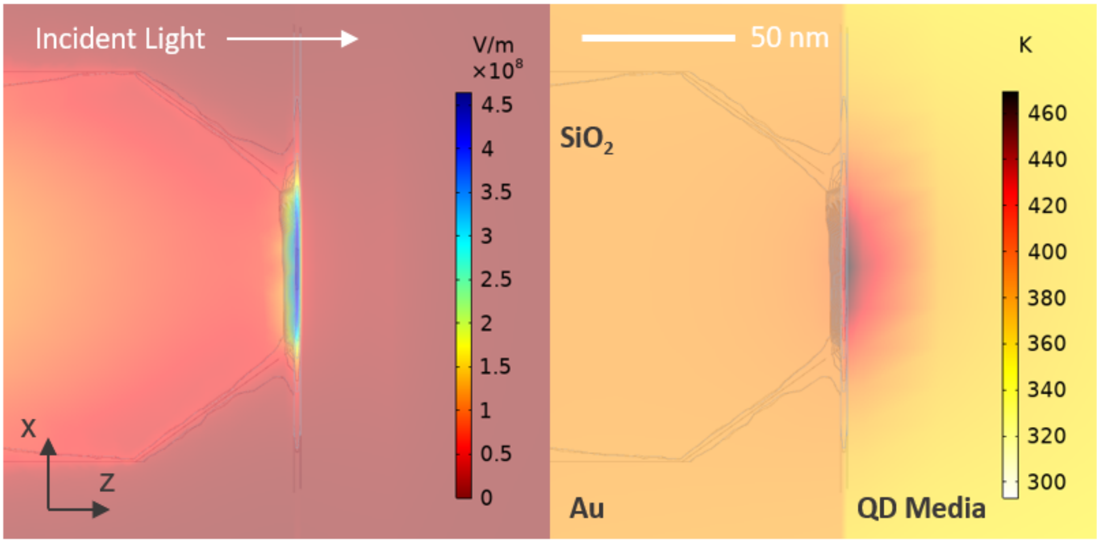

Figure S8 Surface profiles are taken along the upper boundary between the SiO_2-Au interface of the MIM-NFT (identical profiles to Figure 1b). **(a)** The E-field norm is plotted along with contours which depict the regions of maximum power loss used to calculate the temperature of the structure. The contours can be seen to outline the boundary of the SiO_2 insulator layer. **(b)** The temperature is shown to be a maximum in the media with an estimated temperature of 400 K predicted in the Au. One can manipulate the temperature by adding heatsinking material or using metallic alloys in the NFT.

To solve for the maximum temperature of the Au within the NFT, we calculate the steady-state form of the thermal diffusion equation, defined as

$$\frac{\partial T}{\partial t} = D_T \nabla^2 T + Q_s \quad \text{(S5)}$$

where D_T is the diffusion coefficient and the heat source, Q_s, is considered to be dominated by the resistive heating, i.e. power loss, in the system which we extract from the steady-state solution of Eq. S5 and shown in Figure S8.

Table S1: Parameters used in density matrix simulations

**Quantum Dot Parameters**	
Energy level detuning	0 Hz
Radiative decay rate, γ_r	1.2E11 Hz
Dephasing rates	$4.0\gamma_r$
Dipole moment, μ	Aligned in *z*-direction: 5 D, 25 D, and 50 D

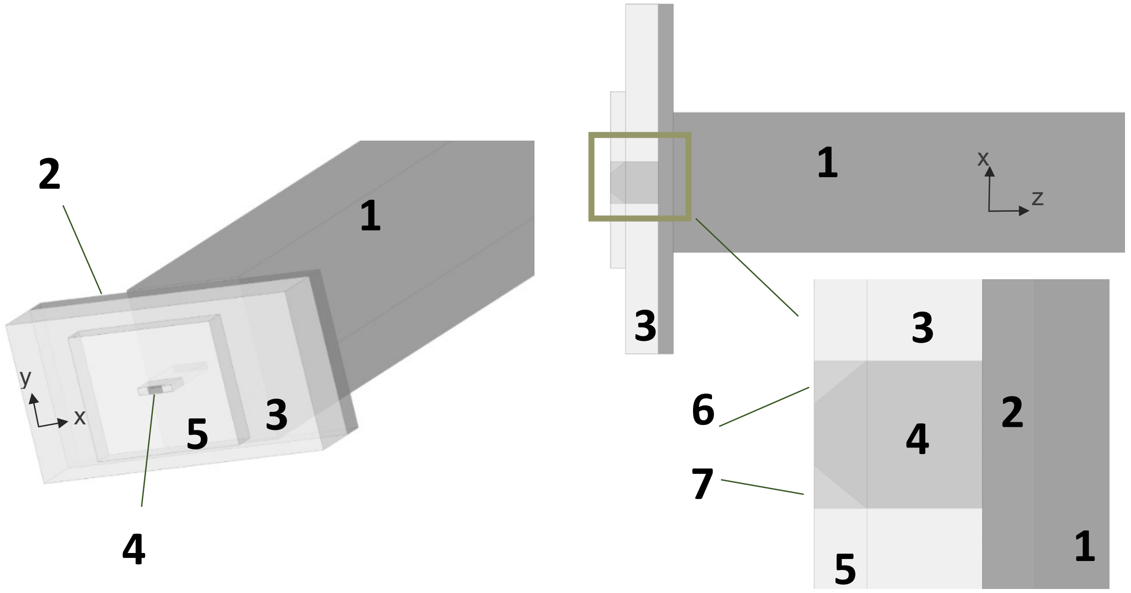

Figure S9. A schematic drawn to scale with numbered values corresponding to the materials and dimension listed in Table S3.

Table S2: Photonic and plasmonic waveguide dimensions.

The numbers in parentheses correspond to labels in Figure S9.

**Waveguide Dimensions (Material, refractive index)**	*length, width, height (x, y, z)*
Slab waveguide (Si_3N_4, 2.0)	400 nm, 400 nm, 2 µm **(1)**
Antireflective trench (SiO_2, n=1.4)	1 µm, 390 nm, 50 nm **(2)**
MIM rear section (Au, 0.06772+5.17625i)	1 µm, 390 nm, 110 nm **(3)**
Insulator (SiO_2)	120 nm (untapered portion), 18 nm, 160 nm **centered at y=11 nm and x=10 nm(4)**
MIM front section (Au)	505 nm, 305 nm, 50 nm, **(5)**
Au tapered region	35 nm, 18 nm, 50 nm **(6, 7)**
**Media Dimensions with x, y plane area of 200×400 nm is considered**	
Air/Lube (z) (2.9+0.12i)	2.0 nm
Multilayered Graphene Layer ε=5.8507+9.1053i with electrical conductivity of 2.7E5 S/m)	29.0 nm
Si-based QD layer (n=3.65)	29.0 nm
*Ag Substrate (0.19+i*5.196), Si Substrate (n=3.42)*	100 nm

**Thermal Parameters used in Diffusion Equation**	Volumetric Heat Capacity (J/(K·m^3)	Thermal Conductivity (W/m·K)
Air/Lube (z)	2E6	1
Ag	2,467,500	429
Si	1,630,300	130
Au	2,569,560	317
SiO_2	2.2E6	1.25
Si_3N_4	2,335,900	30

References

S1. COMSOL MultiphysicsTM v.5.6 www.comsol.com. COMSOL AB, Sweden.

S2. Deinega, A.; Seideman, T. Self-interaction-free approaches for self-consistent solution of the Maxwell-Liouville equations. *Physical Review A* **2014**, *89*, 022501.

S3. Gühne, O.; Seevinck, M., Separability criteria for genuine multiparticle entanglement. *New Journal of Physics* **2010,** *12* (5), 053002.

S4. Sabín, C.; García-Alcaine, G., A classification of entanglement in three-qubit systems. *The European Physical Journal D* **2008,** *48* (3), 435-442.

S5. Sun, G.; Wen, X.; Mao, B.; Chen, J.; Yu, Y.; Wu, P.; Han, S., Tunable quantum beam splitters for coherent manipulation of a solid-state tripartite qubit system. *Nature Communications* **2010,** *1* (1), 51.
